# On gamesmen and fair men: explaining fairness in non-cooperative bargaining games

**DOI:** 10.1098/rsos.171709

**Published:** 2018-02-28

**Authors:** Ramzi Suleiman

**Affiliations:** 1 Triangle Research and Development Center, Kfar-Qari, Israel; 2 Department of Psychology, University of Haifa, Haifa, Israel; 3 Department of Philosophy, Al Quds University, Palestine, Jerusalem, Israel

**Keywords:** bargaining, alternating offers, ultimatum game, Nash equilibrium, subgame perfect equilibrium, Golden Ratio

## Abstract

Experiments on bargaining games have repeatedly shown that subjects fail to use backward induction, and that they only rarely make demands in accordance with the subgame perfect equilibrium. In a recent paper, we proposed an alternative model, termed ‘economic harmony’ in which we modified the individual's utility by defining it as a function of the ratio between the actual and aspired pay-offs. We also abandoned the notion of equilibrium, in favour of a new notion of ‘harmony’, defined as the intersection of strategies, at which all players are equally satisfied. We showed that the proposed model yields excellent predictions of offers in the ultimatum game, and requests in the sequential common pool resource dilemma game. Strikingly, the predicted demand in the ultimatum game is equal to the famous Golden Ratio (approx. 0.62 of the entire pie). The same prediction was recently derived independently by Schuster (Schuster 2017. *Sci. Rep.* **7**, 5642). In this paper, we extend the solution to bargaining games with alternating offers. We show that the derived solution predicts the opening demands reported in several experiments, on games with equal and unequal discount factors and game horizons. Our solution also predicts several unexplained findings, including the puzzling ‘disadvantageous counter-offers’, and the insensitivity of opening demands to variations in the players' discount factors, and game horizon. Strikingly, we find that the predicted opening demand in the alternating offers game is also equal to the Golden Ratio.

## 1. Introduction

Thirty-five years of experimental testing of non-cooperative bargaining theory have repeatedly shown that human subjects do not behave according to game-theoretic predictions. They do not engage as much as the theory prescribes in backward induction [1–4], seldom reach subgame perfect equilibria [1,5–7], show considerable levels of fairness and refrain from exploiting their advantageous strategic positions in the bargaining environment [7,8]. In the ultimatum game [9–11], the subgame perfect equilibrium (SPE) prescribes that the proposer should demand the entire pie, minus an infinitesimally small epsilon to be offered to the responder. This prediction fails miserably in accounting for numerous experimental findings, which indicate that the mean demand in the ultimatum game is approximately 60% of the pie [9,12–15]. Similarly, in bargaining experiments with alternating offers, the SPE is a poor predictor of bargainers' demands and offers [1,2,6,7].

A common finding of experiments on sequential bargaining with shrinking pies is that the opening demands of first players fall somewhere between the equality and the SPE prediction (e.g. [16]). As a consequence, several experimental studies attempted to probe the significance of fairness considerations in ultimatum and sequential bargaining games [8,17–20]. In parallel, theoretical attempts were made in order to incorporate pay-off-interdependent preferences in the game-theoretic model. Such models propose that players' preferences depend not only on the individual monetary pay-off, but also on the pay-offs of others. Ochs & Roth [6] and Bolton [21] suggested that the players' utilities increase in one's own pay-off, and with decrease in the ratio between the pay-offs of the opponent and oneself. The theory of equity, reciprocity and competition (or ERC) [22] posits that, along with pecuniary gain, people are motivated by the ratio of their own pay-offs to the pay-off of others, while inequality aversion (IA) theory [23] assumes that, in addition to the motivation for maximizing own pay-offs, individuals are motivated to reduce the difference in pay-offs between themselves and others, although with greater distaste for having lower rather than higher earnings. Although these modifications yield superior predictions to the standard game-theoretic predictions, they do not qualify as good models of interactive behaviour [2]. For the ultimatum game, the predictions of ERC and IA are uninformative. ERC predicts that the proposer should offer any amount that is larger than zero and less than or equal to 50% of the entire amount, while IA's prediction is non-specific, as it requires an estimation of the players' utility functions, which is not an easy and reliable task. Moreover, both theories are strongly refuted by a three-person ultimatum game designed specifically to test their predictions [24].

Other models of cooperation and fairness, which were proposed to account for the behaviour of human players in bargaining games including the ultimatum game, include models of envy [25], empathy [26,27] and reputation effects [28]. In the case of zero-sum games, like those discussed, the envy model is problematic games, because the effect of ‘envy’ in such games is confounded with the motivation of pure self-interest. For a rational player with utility *u*(*x*), satisfying du(x)/dx>0, where *x* is the player's demand from a pie of size *c*, it immediately follows that du(c−x)/dx=−du(x)/dx <0, with or without envy. In evolutionary games, other factors, such as the heterogeneity of the population [29] and its spatial structure [30–32], were also shown to be important factors in promoting fair behaviours.

A more recent model of cooperation and fairness is based on the concept of ‘cooperative equilibrium’ [33–35]. The model assumes that players have a natural inclination towards cooperation. For the ultimatum game and the Nash bargaining game [36], the model yields a unique cooperative equilibrium for the average behaviour. The model proved successful during the ultimatum game in predicting the minimum offer that would not be rejected by responders (0.2 of the pie) and the range of average offers of approximately 80% of the proposers (between 0.3 and 0.5 of the pie). However, the model does not provide a specific prediction for the mean offer in this game.

Common to all the above-mentioned models is that they presuppose that subjects have some level of other-regarding or benevolent sentiments. By contrast, economic harmony (EH) theory (to be described in §2) prescribes fairness as a predicted outcome reached by rational players, who strive to maximize their own utilities.

An interesting approach to the study of human cooperation and fairness comes from non-equilibrium statistical physics, in particular the Monte Carlo methods and the theory of collective behaviour of interacting particles near phase transition points [37–39]. By treating models of human cooperation as classical spin models, this approach has proved insightful regarding the evolution of cooperation in human societies.

In addition to the failure of the SPE in making quantitative predictions of the demands observed in bargaining experiments, they also fail to provide qualitative explanations for some puzzling results observed in bargaining experiments. In particular, contrary to SPE reasoning, several studies have shown that players in the position of second player who reject opening offers from the first player often give disadvantageous counterproposals, which yield to them less than what they would have received had they accepted the opening offer of the first player [1,6,7,40]. Another puzzling result which contradicts the reasoning of SPE has been reported by Weg *et al.* [7], who found that, in games with unequal discount factors, the stronger of the two players (the one with a higher discount factor) received *less* than 50% of the pie when he or she made the first offer, whereas the weaker player (the one with a higher discount factor) received *more* than 50% of the pie when he or she made the first offer.

In this paper, we use a recently proposed approach to understanding economic interactions [41] for predicting behaviour in bargaining games with alternating offers. The proposed approach, referred to as EH theory, differs from standard game theory in two meaningful aspects: (i) it modifies the utility function by introducing an epistemic factor pertaining to the individual's aspiration level, which depends on the game structure and the player's position in it; and (ii) it abandons the view that bargaining interactions will settle, sooner or later, at an SPE, in favour of the view that interactions will eventually settle at a point at which all the interacting parties are equally satisfied or happy. In formal terms, the proposed approach sways the attention away from the concept of Nash equilibrium—and its refinements—to a new concept of ‘harmony’. While equilibrium is defined as an intersection of players' strategies from which no player is behoved to deviate unilaterally (even when the status quo is disadvantageous and completely unfair to him or her), the harmony solution of a game is defined as the intersection of strategies at which all players are equally satisfied. While a harmony point is not an equilibrium in the formal definition referred to above, it constitutes a critically stable state. The first player can increase her utility by keeping a larger portion of the total amount than the one prescribed by the harmony point, but this will result in decreasing the satisfaction level of the second player, who might reject the unfair offer.

The proposed modification of the utility function is very simple. Instead of assigning the monetary pay-off, *x*, as the argument of the utility function, we assign as an argument the variable *x*/*a*, where *a* is the individual's aspired pay-off in the interaction. As such, the proposed utility function is a measure of the player's level of satisfaction.

It is worth noting that the notion of utility as satisfaction or happiness in the present context is different from Bentham's notion of utility as happiness [42], or its modern adaptation in what is known as ‘happiness studies’ [43]. The problem with these approaches was succinctly elaborated by Benmore [44]. His main criticism was that the definitions of utility in these approaches, and in Rawl's ‘primary goods’ approach, do not allow the comparison between the utilities of different players in the game. In Binmore's words, these approaches end up comparing the utility of Eve from an apple to the utility of Adam from one fig leaf. The definition of utility as satisfaction level in EH theory does not suffer from this problem of interpersonal comparison of utilities, because our definition of utility is based on a dimensionless number, generated for all players by calculating the ratio of a player's actual monetary pay-off to his or her aspired monetary pay-off. As the actual and aspired pay-offs of all players are defined on the same scale of monetary values, it follows that under the linearity of utilities assumption, there is no problem in comparing between different players' utilities. Introducing nonlinearities to the players' utilities does not seem to pose a problem (for more details, see [41]).

Notably, unlike ERC, IA and other pay-off-interdependent theories, EH theory maintains the narrow rationality assumption, such that the utility of a player does not depend on the pay-offs of others. Nonetheless, the theory's predictions, to be detailed hereafter, prescribe that completely self-interested players are bound to behave reasonably fair.

In a recent paper [41], we showed that the proposed theory yields excellent predictions of the offers observed in ultimatum bargaining and the requests in the sequential common pool resource (CPR) dilemma game. Strikingly, the predicted offer in the ultimatum game turned out to be equal to 1 − *φ *≈ 1 − 0.618 ≈ 0.382, where *φ *≈ 0.618 is the famous Golden Ratio, most known for its aesthetic properties [45,46].

In this paper, we extend the theory to bargaining games with alternating offers and fixed discount factors. The remainder of the paper is arranged as follows. Section 2 gives a brief presentation of the theory and summarizes its application to the ultimatum game. Section 3 applies the theory to bargaining games with alternating offers and fixed discounting. Section 4 contrasts the predictions derived in §2 with experimental findings from five studies, which used various (equal and unequal) discount factors and game horizons. Section 5 provides concluding remarks.

## 2. Theory

Consider an economic interaction involving *n* players. Let $S^{i}$ denote the vector of player *i*'s admissible strategies. $S^{i}={\left\{s_{j}^{i}\right\}}_{j=1}^{J^{i}}$, where $s_{j}^{i}$ is strategy *j* of player *i (j* *=* 1, 2, … *J_i_*, *i* = 1, 2, …, *n*). We define the subjective utility of each player *i* as follows:

2.1 $$u_{i}(..)=u_{i}\left(\frac{r_{i}}{a_{i}}\right),$$

where *r_i_* is player *i*'s actual pay-off, *a_i_* is his or her aspired pay-off and *u*(..) is a bounded non-decreasing utility function with its argument. For simplicity, we assume that *u*(0) = 0 and *u*(1) = 1. Note that, in social–psychological terms, the aforementioned definition implies that each player puts an upper limit on his or her greed.

It is reasonable to assume that the aspiration levels, *a_i_* (*i* *=* 1, 2, …, *n*), depend on the interaction between the individuals' distinctive personalities, the game structure and the players' positions in the game. Ideally, one would opt to measure the individuals' aspirations in situations which resemble as much as possible their respective positions in the investigated game environment. In the absence of empirical measures, as a first-order estimation, we shall make plausible assumptions regarding the aspiration levels of players in the discussed games.

A point of harmony in an interaction is defined as an intersection point of strategies, played by the *n* interacting individuals, at which the utilities of all players, as defined above, are equal. In formal terms, a point of harmony in an interaction between *n* players is a vector of outcomes r* = ($r_{1}^{*},r_{2}^{*},r_{3}^{*},\ldots .,r_{n}^{*}$) for which the subjective utilities of all *n* players' outcomes satisfy

2.2 $$u_{i}\left(\frac{r_{i}^{*}}{a_{i}}\right)=u_{j}\left(\frac{r_{j}^{*}}{a_{j}}\right)\;\text{For all}\;i\;\text{and}\;j.$$

Assuming linear utilities, equation (2.2) becomes

2.3 $$\frac{r_{i}^{*}}{a_{i}}=\frac{r_{j}^{*}}{a_{j}}\;\text{For all}\;i\;\text{and}\;j.$$

One might ask how could players infer about other players' aspirations? And why should they care about others' satisfaction levels? The answer is twofold. First, by using their theory of mind, players can imagine themselves in the positions of others and estimate their levels of aspiration. As an example, a proposer in the ultimatum game would take the perspective of the responder and ask himself: ‘what would I aspire for, had I been in the proposer's position’. Second, in repeated games, the estimates of others' aspirations, conjointly with the objective aspirations, are expected to evolve through learning. As in the case of the Nash equilibrium and its refinements, in repeated interactions, players are not expected to ‘solve’ the game and play their harmonious strategies. Rather, it is conjectured that harmonious strategies can emerge through some processes of learning and adaptation. In our epistemic theory, adaptation processes are expected to act interdependently on each individual's decisions and aspirations. A valuable insight into the coevolution of strategies and aspiration levels comes from simulation studies of both reinforcement learning [47,48] and evolutionary ultimatum games [28,49,50]. For example, Nowak *et al.* [28] demonstrated that when individuals obtain some information on which offers have been accepted by others in previous encounters, responders in the game learn to maintain high aspiration levels. As explained by the authors, responders who lower their aspiration levels (low demands) increase the chance of receiving reduced offers in subsequent encounters. By contrast, by maintaining high aspiration levels (high demands), although costly, they gain a reputation as individuals who insist on a fair offer.

## 3. Bargaining games with alternating offers

Our main objective in this paper is to apply the theory of EH to bargaining games with alternating offers, first proposed and analysed by Rubinstein [51]. For better readability, we shall focus on games with fixed discounting factors. In a typical game of this type, one party proposes a division of the ‘pie’, which the other must accept or reject. If the proposal is accepted, the game ends, and the parties receive their shares of the pie. If the proposal is rejected, the game continues for another period, and the right to make a proposal goes to the second player. If the first player accepts the proposal of the second player, the game ends with the two receiving their respective shares, but if the first player rejects the proposal, the right to propose a division goes back to the first player. The value of the pie shrinks during each period. The shrinkage from one period to another could be equal or different for each player. In a game with a finite horizon, if agreement is not reached in a fixed number of periods, both parties receive zero pay-offs. In the infinite horizon game, the alternation of offers continues until an agreement is reached, or alternatively, until the pie shrinks to zero.

The solution for the finite game could be worked out using the backward induction method. For the infinite horizon game, backward induction does not apply. For such games, Rubinstein [51] proved the existence of a unique SPE, which depends on the form of the pie shrinkage. For a game with fixed discount factors $\delta_{1}\text{ and }\delta_{2}$ ($0<\delta_{1},\delta_{2}<1$), the only SPE prescribes that the game should end in the first period, with player 1 demanding $1-\delta_{2}/(1-\delta_{1}\delta_{2})$. For the special case $\delta_{1}=\delta_{2}=\delta$, the aforementioned solution reduces to 1/(1 + *δ*) (0 < *δ* < 1).

To derive the harmony solution for bargaining games with alternating offers, infinite horizon and fixed discounting, consider a game in which the pie shrinks after each period with discount factors *δ*_1_ and *δ*_2_, for players 1 and 2, respectively (0 < *δ*_1_, *δ*_2_ < 1). Suppose that the players have completed *n −*1 periods without reaching an agreement. For *n*, an odd number, it is the turn of player 1 (hereafter P1) to make an offer of how to divide a pie, which has shrunk from 1 monetary unit (MU) in period 1 to ${\delta_{1}}^{n-1}$ for her and to ${\delta_{2}}^{n-1}$ for player 2 (hereafter P2). Suppose that P1 demands a portion of *x*_1,*n*_ and offers a portion of 1 − *x*_1*,n*_ to P2. A harmonious agreement is reached in period *n* if the following condition is satisfied:

3.1 $$\frac{{\delta_{1}}^{n-1}x_{1,n}}{a_{1,n}}=\frac{{\delta_{2}}^{n-1}\;(1-x_{1,n})}{a_{2,n}}\;n=1,3,5,\ldots$$

where *a*_1,*n*_ and *a*_2,*n*_ are the aspiration levels of P1 and P2 at period *n*, respectively. If P1 is perfectly rational, then she would aspire for the entire pie; i.e. $a_{1,n}={\delta_{1}}^{n-1}$. As in the case of ultimatum bargaining [41], we consider two intuitive assumptions regarding the aspiration level of P2: (i) that he or she aspires for absolute equality, i.e. $a_{2,n}={\delta_{2}}^{n-1}\text{/}2$; and (ii) that he or she aspires for proportional equality or equity, i.e. $a_{2,n}={\delta_{2}}^{n-1}x_{1,n}$. Note that both the equality norm and the equity norm are representatives of canonical ethics. The first reflects an aspiration for equality, i.e. that the first player would offer an equal split. The second norm reflects a proportional equality norm, i.e. that the first player grants the responder an amount relative to what she keeps to herself, and she keeps for herself an amount relative to the entire amount. In other words, the second player would hope that, in relative terms, the first player treats her as she treats herself. The proportional equality second rule is congruent with the teachings of Abrahamic and other religions, which advocate that individuals should treat others as they treat themselves (for more details, see [52]).

In the following, we derive the solution of the bargaining game under each of the equality and proportional equality (equity) norms. We show that the two predictions are quite close to each other.

### 3.1. P2 aspires for absolute equality

Under this assumption, we have $a_{2,n}={\delta_{2}}^{n-1}\text{/}2$. Substitution in equation (3.1) gives

3.2 $$\frac{{\delta_{1}}^{n-1}x_{1,n}}{{\delta_{1}}^{n-1}}=\frac{{\delta_{2}}^{n-1}\;(1-x_{1,n})}{{\delta_{2}}^{n-1}\text{/}2}\;n=1,3,5,\ldots$$

which after simplification yields

3.3 $$x_{1,n}=2(1-x_{1,n})\;n=1,3,5,\ldots$$

or

3.4 $$x_{1,n}=\frac{2}{3}\;n=1,3,5,\ldots$$

which corresponds to a division of ($(2/3){\delta_{1}}^{n-1}$, $(1\text{/}3){\delta_{2}}^{n-1}$) for P1 and P2, respectively. As *δ*_2_ < 1, P2 is better off accepting P1's opening offer and receiving 1/3 MU, while P2 receives 2/3 MU, which is also optimal for her.

### 3.2. P2 aspires for equity

Throughout the paper, we shall stick to the proportional equality interpretation; i.e. we shall assume that P2 adheres to social comparison and aspires to be offered as much as P1 demands for herself. However, the theoretical results for the equality assumption could be worked out similarly. In general, they yield quite close predictions to the ones to be derived hereafter. Under the proportional equality assumption, we have $a_{2,n}={\delta_{2}}^{n-1}x_{1,n}$. Substitution in equation (3.1) gives

3.5 $$\frac{{\delta_{1}}^{n-1}x_{1,n}}{{\delta_{1}}^{n-1}}=\frac{{\delta_{2}}^{n-1}\;(1-x_{1,n})}{{\delta_{1}}^{n-1}\;x_{1,n}}\;n=1,3,5,\ldots$$

which yields

3.6 $${x_{1,n}}^{2}+{\left(\frac{\delta_{2}}{\delta_{1}}\right)}^{n-1}x_{1,n}-{\left(\frac{\delta_{2}}{\delta_{1}}\right)}^{n-1}=0\;n=1,3,5,\ldots$$

Define $\delta_{1}\text{/}\delta_{2}$ = *R*. For positive *x_n_* equation (3.6) solves for

3.7 $$x_{1,n}=\left(\frac{\sqrt{1+(4\text{/}R^{n-1})}-1}{2}\right)\;R^{n-1}\;n=1,3,5,\ldots$$

Furthermore, the demand of P1 and transfer to P2 are, respectively,

3.8 $$d_{1,n}={\delta_{1}}^{n-1}x_{1,n}={\delta_{1}}^{n-1}\;R^{n-1}\left(\frac{\sqrt{1+\frac{4}{R^{n-1}}-1}}{2}\right)$$

and

3.9 $$t_{1,n}={\delta_{2}}^{n-1}\;(1-x_{1,n})={\delta_{2}}^{n-1}\left(1-R^{n-1}\left(\frac{\sqrt{1+(4\text{/}R^{n-1})}-1}{2}\right)\right).$$

For the sake of simplicity, first we discuss the special case of equal discounts (*R* = 1), and then we turn to the general case of unequal discounts (*R* ≠ 1).

#### 3.2.1. Equal discount factors (*R* = 1)

For *δ*_1_ = *δ*_2_ = *δ* (*R* = 1), equations (3.7)–(3.9) reduce to

3.10 $$\begin{array}{rl} x_{1,n} & =\frac{\sqrt{5}-1}{2}=\varphi , \end{array}$$

3.11 $$\begin{array}{rl} d_{1,n} & =\varphi \;\delta^{n-1}\approx 0.618\;\delta^{n-1} \end{array}$$

3.12 $$\begin{array}{rl} \mathrm{and}\;\;t_{2,n} & =(1-\varphi )\;\delta^{n-1}\approx 0.382\;\delta^{n-1}, \end{array}$$

where *φ* is the Golden Ratio (*φ* ≈ 0.618).

As both discount factors are positive and less than 1, the maximal pay-off for P1 and P2, while maintaining harmony, is achieved in period *n* = 1. Setting *n* = 1 in equations (3.11) and (3.12), the respective demand of P1 and transfer to P2 become

3.13 $$d_{1,1}=\varphi \;\delta^{0}=\varphi \approx 0.618$$

and

3.14 $$t_{2,1}=(1-\varphi )\;\delta^{0}=(1-\varphi )\approx 0.382.$$

Thus, for the case of equal discount factors, EH predicts that the game should end in period 1, with P1 making an opening demand of *d*_1,1_ = *φ* ≈ 0.618 of the pie and P2 accepting a share of (1 − *φ*) ≈ 0.382. Strikingly, this prediction is identical to the one derived by the theory for the one-period ultimatum game and which was shown to account successfully for the approximately 0.6–0.4 divisions reported in numerous ultimatum experiments (for details, see [41]).

Now suppose that, for some reason, agreement is not reached in period 1. For example, the first player might give a low offer that the second player rejects. In this case, the roles switch so that it is the turn of P2 to make a demand from a pie of size *δ.* Assume that P2 demands a portion of *d*_2,2_ and offers a portion of 1 − *d*_2,2_ to P1. A harmonious agreement will be reached again at the Golden Ratio, now with P2 demanding a portion equalling *d*_2,2_ = *φ δ ≈ *0.618 *δ* and offering a portion of (1 − *φ*) *δ *≈ 0.382 *δ* to P1. For the case in which *φ δ* > 1 − *φ*, or δ>(1−φ)/φ, a player in the position of P2 who wants to maximize her harmony pay-off will be better off if she rejects P1's opening offer and demands *d*_2,2_ = *φ δ ≈ *0.618 *δ* in period 2. As *φ*^2^ + *φ* − 1 = 0, substituting 1 − *φ* = *φ*^2^ in the above inequality enables it to be rewritten as *δ* > *φ*. A player in the position of P1 who can make a one-step backward induction could end the game in agreement in period 1 by lowering his opening demand from the harmony demand of *φ* to 1 − *φ δ*.

The same analysis could be extended to more than two periods; however, we know based on ample evidence that players are limited in their cognitive ability to make backward induction [1–4]. Thus, we suffice by making predictions for the opening proposal by P1 and counterproposal by P2, in case she rejects P1's proposal. For the first two periods, EH predictions for the case of equal discount factors is simple and could be summarized as follows:

1. For *δ* ≤ *φ*, and all game horizons, the bargaining should end with agreement in the first period, with P1 demanding (and receiving) a portion of *d*_1,1_ = *φ *≈ 0.618 (and P2 receiving a portion of 1 − *φ* ≈ 0.382).
2. For *δ* > *φ*:
  (a) The game might end with agreement in the first period, with P1 demanding and receiving *d*_1,1_ = 1 − *φ δ* (and P2 receiving *φ δ*) or
  (b) The game might end with agreement in the second period, with P2 demanding and receiving *d*_2,2_ = *φ δ* (and P1 receiving (1 − *φ*) *δ*).
3. For *δ* ≤ *φ*, if the game continues to the second period, then the demand of P2 (*φ δ*) will be less than or equal to the offer proposed by P1 and rejected by her in the first period (1 − *φ*).

#### 3.2.2. Unequal discount factors (*R ≠ *1)

For the general case of unequal discount factors, the term for *x*_1,*n*_ is depicted in equation (3.7). The opening partition by P1 could be calculated by substituting *n* = 1, which yields

3.15 $$x_{1,1}=\left(\frac{\sqrt{1+(4/R^{0})-1}}{2}\right)\;R^{0}=\frac{\sqrt{5}-1}{2}=\varphi .$$

The demand by P1 and his offer to P2 are, respectively, *φ* and 1 − *φ.* Thus, the harmony opening demand derived for the equal discounts case holds for the general case of all *δ*_1_ and *δ*_2_ values.

**Figure 1.**
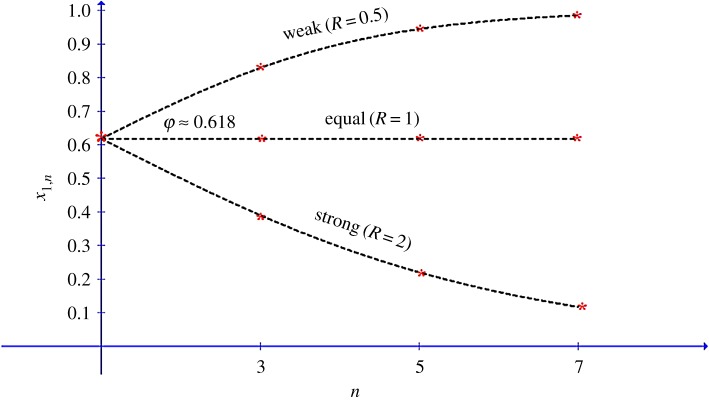
The partition *x*_1*,n*_ by strong, equal and weak player in the role of P1 as a function of period number *n*.

Weg *et al.* [7] distinguish between games with ‘strong’ P1 (*δ*_1_ > *δ*_2_, or *R* > 1), ‘equal’ discounts (*δ*_1_ = *δ*_2_, or *R* = 1) and ‘weak’ P1 (*δ*_1_ < *δ*_2_, or *R* < 1). Figure 1 depicts *x*_1,*n*_ from equation (3.7), as a function of *n*, for *n* = 1, 3, 5, 7, and for ‘strong’, ‘equal’ and ‘weak’ P1, with *R* = 2, 1 and 0.5, respectively. As shown in the figure, the predicted harmony partition, *x*_1,1_ in the first period, is equal to the Golden Ratio regardless of the relative strength of the two players. Interestingly, the harmony partition by a ‘strong’ P1 player is predicted to decrease with the number of periods, *n* (*n* = 1, 3, 5,…), while for a ‘weak’ P1 player, the harmony partition is predicted to increase with *n*. The same unexpected result applies to the harmony partitions by P2 when it is her turn to propose. Thus, for games which continue for more than one period, the theory predicts that the mean pay-off for strong players will be less than that for weak players.

Focusing only on the first two periods of the game, a similar analysis to the one performed for the equal discounts case yields the following predictions:

1. For *δ*_2_ ≤ *φ* ≈ 0.618 and all *δ*_1_ values, the game should end with agreement in the first period, with P1 demanding (and receiving) a Golden Ratio portion of the pie (*d*_1,1_ = *φ* ≈ 0.618) and P2 receiving 1 − *φ* ≈ 0.382.
2. For *δ*_2_ > *φ* ≈ 0.618, and all *δ*_1_:
  (a) The game might end in the first period, with P1 demanding and receiving *d*_1,1_ = 1 − *φ δ*_2_ ≈ 1 − 0.618 *δ*_2_ (and P2 receiving $\varphi \delta_{2}\approx 0.618\;\delta_{2}$). In other words, for this case, the theory predicts a linear decline in the demand of P1, with the slope equalling 0.618 or
  (b) The game might end in the second period, with P2 demanding and receiving *d*_2,2_ = *φ δ*_2_ ≈ 0.618 *δ*_2_ (and P1 receiving (1 − *φ*) $\delta_{1}\approx 1-0.618\;\delta_{1})$.
3. For *δ*_2_ ≤ *φ*, if the game continues to the second period, then the demand of P2 (*φ δ*_2_) will be less than or equal to the offer proposed by P1 and rejected by her in the first period (1 − *φ*).
4. If the game continues beyond period 1, then with the progression of the game, strong players will reduce their demanded portions of the pie, whereas weak players will increase their demanded portions. Thus, the theory predicts that, in such games, the mean pay-off for the strong player will be less than that for the weak player.

**Figure 2.**
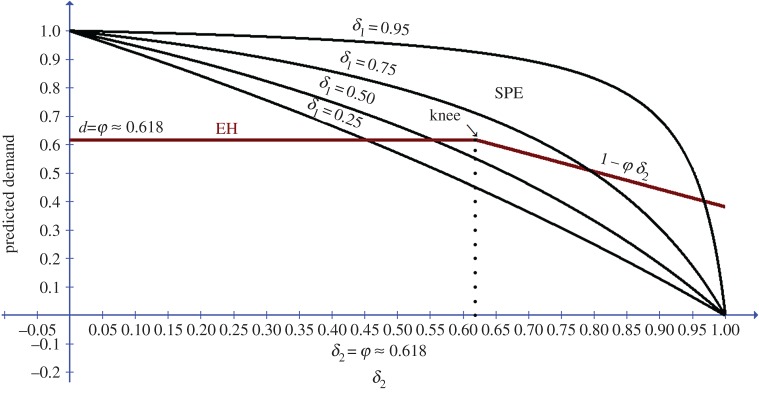
Predicted opening demands of P1 by EH and SPE as functions of the discount factor of P2.

For games ending in agreement in period 1, figure 2 depicts the opening demands predicted by EH and SPE for the selected *δ*_1_ value as functions of *δ*_2_.

As could be seen in figure 2, the predicted harmony demand by P1 in the first period, as a function of *δ*_2_, exhibits a non-monotonicity (or ‘knee’) effect. For values of *δ*_2_ which are less than or equal to the Golden Ratio, P1 should demand a portion of the pie equalling the Golden Ratio. For values of *δ*_2_ which are larger than the Golden Ratio, the harmony demand of P1 in the first period is predicted to decline linearly with delta, with the slope equalling the Golden Ratio.

## 4. Theory testing

We tested the above predictions using data from five studies, which employed a variety of discount factors and game horizons. The investigated experiments, together with their parameters, are depicted in table 1. The five experiments listed in the table, although far from being comprehensive, are fair representatives of studies using the fixed discounting mechanism. As can be seen in the table, the investigated studies cover a range of game lengths (*T*) and discount factors, with some discounts used in more than one study. The Binmore *et al.* [2] study included eight discount factors, five of which are lower than the predicted Golden Ratio threshold and three are higher than the threshold. This design is ideal for testing the predicted discontinuity effect. The last two studies in the table, by Ochs & Roth [6] and Weg *et al.* [7], enable us to test the performance of the theory for the general case of unequal discount factors, including its counterintuitive prediction regarding the effect of the players' relative strengths on their partitions of the pie.

**Table 1.** Experimental conditions of five investigated studies.

study	pie size	discount factors	horizon (*T*)
		equal (*δ*)	
Binmore *et al.* [40]	100 pennies	0.25	2
Neelin *et al.* [1]	$5 and $10	0.25, 0.5, 0.34	2, 3, 5
Binmore *et al.* [2]	100 points pay-offs determined by a roulette	0.2, 0.3, 0.4, 0.5, 0.6, 0.7, 0.8, 0.9	2
		unequal (*δ*_1_, *δ*_2_)	
Ochs & Roth [6]	$30	(0.4, 0.4), (0.6, 0.4), (0.6, 0.6), (0.4, 0.6)	2, 3
Weg *et al.* [7]	60 New Israeli Shekels	(0.9, 0.5), (0.67, 0.67), (0.5, 0.9)	infinite

In the following subsections (§§4.1—4.5), we shall test the point predictions of the theory for each study separately. In §4.6, we shall test the predicted disadvantageous counterproposal by the second player. Moreover, the studies by Binmore *et al.* [2] and Weg *et al.* [7] provide us with the opportunity to test the predicted ‘knee’ effect at *δ*_2_ ≈ 0.6.

### 4.1. The Binmore *et al.* [40] study

Binmore *et al.* [40] examined the SPE using a two-stage game. The SPE prescribes that P1 should make an opening demand in the range of 74–76 pence (p.), and P2 should accept any opening demand of 74 pence or less, because he or she cannot do better by rejecting it, even if he or she obtains the entire cake in the second stage. Binmore *et al.* [40] conducted two identical experiments, except that the participants who filled the role of P1 in the second experiment were the subjects who filled the role of P2 in the first experiment. Comparison between the results of the two experiments revealed a marked change in the demands of players in the role of P1, who had previously experienced being in the role of P2. In the first experiment, out of 82 opening demands, approximately 55% were 50 p. or less, with only approximately 20% with demands close to the SPE. In the second experiment, the results shifted a period. Out of 81 opening demands, only approximately 24% were 50 p., while approximately 62% were close to the SPE. The researchers concluded rightfully that the tendency to ‘play fair’ in the first experiment shifted towards a strong tendency to play ‘like a game theorist’ in the second experiment. Notwithstanding, we believe that this result could hardly be considered as support for the SPE prediction, because the first players in the second experiment must have gained experience from having previously been in P2's role to help them put themselves in the shoes of P2 in the second experiment and to strategize about what minimal offers a player in P2's position in the second experiment might be willing to accept. In any case, for possible contamination of the results of the second experiment, we looked only at the results of the first experiment. The results of the first experiment revealed that 85% of the games ended with agreement in period 1. The mean opening demand of P1 was 60 p., or 0.6 of the entire pie. As *δ*_2_ (=0.25) is less than the critical value *φ* = 0.618, EH theory predicts that agreement should be reached in the first period, with P1 demanding the Golden Ratio (*d*_1,1_ = *φ *≈ 0.618), or approximately 62 p. The absolute prediction error of EH is |60−62/60|×100=3%. For comparison, the prediction error of SPE is |60−75/60|×100=25%.

### 4.2. The Neelin *et al.* study

Neelin *et al.* [1] replicated the study by Binmore *et al.* [40], but varied the number of periods in the between-subjects design. The study included two experiments: one experiment with a small pie size of *M* = $5 and *n* = 2, 3 and 5 periods, and another experiment with five periods and three times the pie size (*M* = $15).

The design of the first experiment is depicted in table 2. The values to be divided in each period were set to ensure that, in all three games, the SPE model predicts that agreement should be reached in the first period, with P1 demanding $3.75 (and offering $1.25 to P2).

**Table 2.** Value in $ to be divided in each period ([1], Exp. 1).

period number	two periods	three periods	five periods
1	5.00	5.00	5.00
2	1.25	2.50	1.70
3	−	1.25	0.58
4	−	−	0.20
5	−	−	0.07

Note that, for the game with five periods, one might assume that, under the time pressure of the experiment, the players might be unable to work out the backward induction properly and that they might perceive the game as one having many periods. Extrapolating the five-period game ad infinitum and applying the Rubinstein solution [51] for a game with infinite horizon gives 1/(1 + *δ*) ≈ 1/(1 + 0.34) ≈ $3.73, which is only 2 cents less than the exact solution of $3.75.

The results of the first experiment by Neelin *et al.* [1] revealed that the percentage of games which ended in agreement in period 1 were 87%, 84% and 86% for conditions *T* = 2, 3 and 5, respectively. The results of the two-period condition replicated those reported by Binmore *et al.* [40]. Fifteen out of 40 subjects demanded exactly the Stahl/Rubinstein division [51,53], which gave them $3.75, and 18 subjects demanded between $3.50 and $3.74. By contrast, the results of the three- and five-period conditions strongly refuted the prediction of the Stahl/Rubinstein model. In the game with *T* = 3, 28 out of 40 subjects demanded $2.50, and four others demanded slightly less than $2.50. Only three subjects demanded between $3.50 and $3.74, and none demanded the exact equilibrium prediction of $3.75. The results of the game with *T* = 5 showed that 33 out of 40 subjects demanded between $3.00 and $3.49. Two subjects demanded $2.50, two others demanded between $3.50 and $3.74, and none demanded the exact SPE prediction of $3.75. While the demands in the three-period game could be interpreted as supportive of the equality principle, the demands in the five-period game accord neither with the equality principle nor with the SPE prediction.

#### 4.2.1. Economic harmony predictions

As all three discount factors implemented in the game are less than the critical value *δ* = *φ *≈ 0.618, EH predicts that agreement should be reached in the first period, with P1 demanding a portion of the pie equalling *φ *≈ 0.618. For a pie size of $5, the predicted opening demand of P1 is 0.618 × $5 = $3.09. Calculating the observed mean demands in the three games yields 3.63, 2.57 and 3.27 for the two-, three- and five-period games, respectively (mean = 3.16, s.d. = 0.44). The mean (absolute) prediction error for EH is equal to |(3.16−3.09)/3.16| × 100 = 2.2%. In comparison, the SPE prediction error is |(3.16−3.75)/3.16| × 100 = 18.7%.

In the second experiment, Neelin *et al.* [1] tested the robustness of the behaviour observed in the first experiment by increasing the level of bargaining experience and the amount at stake. Thirty subjects participated in the experiment. They played the five-period game four times, once for practice and three times for cash. The pie size in all games was $15. The SPE for all games is $11.25. The results of the second experiment revealed an impressive level of consistency in the participants' behaviour across the three experimental games. There was no evidence of movement towards the SPE, and experience did not change the opening demands. Almost all bargains ended with agreement in the first period. Seventy per cent of the opening demands of P1 were between $9.50 and $10.00. Eleven to twelve subjects demanded $9.50–$10.00. No demands close to the SPE demand of $11.25 were observed. Comparison with the results of the five-period condition in the first experiment showed that changing the amount at stake did not affect the demand significantly.

#### 4.2.2. Economic harmony prediction

Calculating the mean demands for the three experimental games yields $9.83, $9.49 and $9.49 for games 1–3, respectively, with a grand mean of $9.60. As *δ* = 0.34 < *φ *≈ 0.618, EH predicts that agreement should be reached in the first period, with the first player demanding 0.618 × $15 = $9.27. The prediction errors in absolute terms for EH and the SPE predictions are, respectively, |(9.60−9.27)/9.60| × 100 = 3.4% and |(9.60−11.25)/9.60| × 100 = 17.2%. If we look only at the behaviour in the last game, at which maximum experience has been gained, the prediction errors of EH and SPE become 2.3% and 18.6%, respectively.

### 4.3. The Binmore *et al.* [2] study

Binmore *et al.* [2] reported experiments with one-stage and two-stage alternating offers bargaining games, with various discount factors. Their main purpose was to examine whether, given the pay-off-interdependent preferences, players will respect backward induction. Their findings were strong in showing that players' behaviour systematically violates both subgame and truncation consistency.

**Figure 3.**
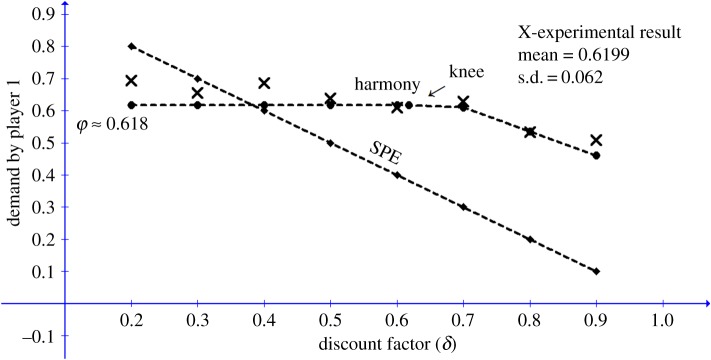
Experimental and theoretical opening demands by P1 for the Binmore *et al.* [2] study, as functions of the discount factor *δ.*

Game III in the study included two periods, played under eight discount factors (*δ* = 0.2, 0.3, 0.4, … , 0.8, 0.9). This game provides ideal data for testing the ‘knee’ effect at *δ*_2_ = 0.6, predicted by EH theory. The five lower discount factors (*δ* = 0.2, 0.3, …, 0.6) are below the threshold point of 0.618 predicted by the EH model, while the remaining three discount factors (*δ* = 0.7, 0.8, 0.9) are above the threshold. For the lower five *δ* conditions, the theory predicts that agreement should end in period 1, with P1 demanding ≈ 0.618 of the pie. For the highest three *δ* conditions, the predicted opening demand by P1 is 1 − *φ δ *≈ 1 − 0.618 *δ*. Table 3 and figure 2 depict the mean demands under each *δ* condition, together with the predictions of the EH and SPE models. The mean demand across the eight conditions is equal to 0.6199 (s.d. = 0.062). A linear regression of the observed demand on the predicted demand by EH yields an *R*^2^ of ≈0.82. The superiority of the EH model over the SPE and the equality models is quite evident. The prediction error for the harmony solution is $\left(1\text{/}n)\Sigma_{n}(|d_{o}-d_{p}|/d_{o}\right)$ × 100 = 7.3%. The prediction errors of the equality and the SPE models are, respectively, 17.7% and 36.4%. Strikingly, the mean demand exhibits a ‘knee’ effect at *δ* = 0.6, as predicted by the harmony model (figure 3).

**Table 3.** Experimental and theoretical opening demands by P1 for each *δ* condition.

	discount factor (*δ*)							
demand	0.2 (*n* = 50)	0.3 (*n* = 50)	0.4 (*n* = 50)	0.5 (*n* = 50)	0.6 (*n* = 50)	0.7 (*n* = 50)	0.8 (*n* = 50)	0.9 (*n* = 50)
experimental	0.693	0.655	0.686	0.639	0.610	0.628	0.534	0.509
harmony prediction	0.618	0.618	0.618	0.618	0.618	0.567	0.506	0.444
SPE prediction	0.80	0.70	0.60	0.50	0.40	0.30	0.20	0.10

### 4.4. The Ochs & Roth study

Ochs & Roth [6] tested the SPE model using either equal or unequal discount factors. The combinations of the discount factors used in the study were (*δ*_1_ = 0.4, *δ*_2_ = 0.4), (*δ*_1_ = 0.6, *δ*_2_ = 0.4), (*δ*_1_ = 0.6, *δ*_2_ = 0.6) and (*δ*_1_ = 0.4, *δ*_2_ = 0.6). Each condition was tested in games with horizons equalling *T* = 2 and *T* = 3. Table 4 depicts the mean proportion of P1's opening demand out of the entire pie under the four combinations of the tested discount factors. The SPE predictions are calculated for each condition by the method of backward induction. For games with *T* = 2, the SPE in period 1 is equal to $1-\delta_{2}$, while for games with *T* = 3, it is equal to 1 − (*δ*_2_ − ${\delta_{1}}^{2}$). As for all the tested conditions *δ*_2_ < *φ*, EH harmony predicts that P1's opening demand is equal to *φ* ≈ 0.618.

**Table 4.** Experimental and theoretical opening demands of P1 for each experimental condition.

		*δ*_2_ = 0.4			*δ*_2_ = 0.6		
	horizon (*T*)	experimental	harmony	SPE	experimental	harmony	SPE
*δ*_1_ = 0.4	2	0.561	0.618	0.40	0.511	0.618	0.60
	3	0.566		0.76	0.537		0.560
*δ*_1_ = 0.6	2	0.509		0.40	0.538		0.60
	3	0.532		0.96	0.536		0.76

**Figure 4.**
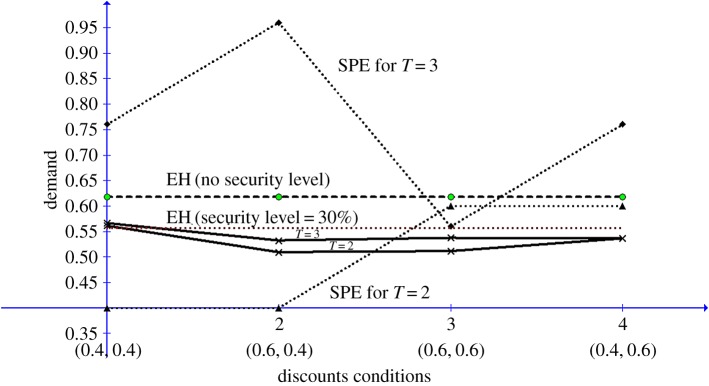
Experimental and theoretical opening demands by P1 for the Ochs & Roth [6] study as functions of the discount factors conditions.

The experimental and theoretical results are depicted graphically in figure 4. As noted by the authors, the mean opening demands are quite uniform across the tested (*δ*_1_, *δ*_2_) conditions. This unexpected result in nicely captured by EH, which makes the same prediction for all investigated conditions. Moreover, as predicted by EH, the effect of the game's horizon on P1's opening demand is quite insignificant. The mean prediction error of the EH model is 15.4%, compared with a prediction error of 30% for the SPE. Moreover, as shown in table 4, for horizon *T* = 2, the differences between the observed demands and the predicted demands by EH, for the conditions (0.6, 0.4) and (0.6, 0.6), are almost equal. The same holds for the differences for *T* = 3, between the observed and predicted opening demands under the conditions (0.6, 0.4), (0.6, 0.6) and (0.4, 0.6). This implies that introducing a moderate level of risk aversion to the player's utility function, or relaxing the assumption that P1 aspires for the entire pie, could result in significant improvement in EH prediction power. As an example, if we assume that P1 has a security level (cf. [54]) of 0.3 (meaning that he aspires for 0.70 of the pie), then for *n* = 1, equation (3.6) becomes

4.1 $${x_{1}}^{2}+0.7\;x_{1}-0.7=0,$$

which solves for

4.2 $$x_{1}\approx 0.557.$$

For this case, the prediction error of EH decreases from 15.4% to only 4.6%.

### 4.5. The Weg *et al.* study

Weg *et al.* [7] investigated a two-person bargaining game with alternating offers and infinite horizon. They reported two experiments in which the discount factors of the players were manipulated in a between-subjects design, such that the discount factor for P1 was more than, equal to or less than the discount factor of P2. The authors termed the three conditions as strong (S), equal (E) and weak (W). In all conditions, the pie to be split was equal to 60 Shekel (approx. $16). In the first experiment, the discount factors' conditions were (*δ*_1_ = 0.9, *δ*_2_ = 0.5), (*δ*_1_ = 0.67, *δ*_2_ = 0.67) and (*δ*_1_ = 0.5, *δ*_2_ = 0.9), and in the second, the conditions were (*δ*_1_ = 0.50, *δ*_2_ = 0.17), (*δ*_1_ = 0.17, *δ*_2_ = 0.17) and (*δ*_1_ = 0.17, *δ*_2_ = 0.50). Here, we investigate only the results of the first experiment, because the values chosen for *δ*_2_ in this experiment (0.5, 0.67, 0.9) allow us to test the predicted discontinuity effect. This prediction prescribes that, for *δ*_2_ = 0.5, P1 should demand *φ *≈ 0.618 of the pie, while for the other two conditions (*δ*_2_ = 0.67 and *δ*_2_ = 0.9), the demands of P1 are predicted to be equal to 1 − *φ δ*_2_. For *δ*_2_ = 0.67, the predicted demand is 1 − 0.618 × 0.67 = 0.586, and for *δ*_2_ = 0.9, the predicted demand is 1 − 0.618 × 0.9 = 0.444. The SPE prediction is calculated using the Rubinstein solution, which prescribes that the opening demand of P1 is equal to $\left(1-\delta_{2})/(1-\delta_{1}\delta_{2}\right)$. For the three tested conditions (*δ*_1_ = 0.9, *δ*_2_ = 0.5), (*δ*_1_ = 0.67, *δ*_2_ = 0.67) and (*δ*_1_ = 0.5, *δ*_2_ = 0.9), the SPE predictions are 0.909, 0.599 and 0.181, respectively. The results of the experiment revealed that, on average, 79.4% of the games ended with agreement in period 1. The mean proportional opening demands were 0.50, 0.48 and 0.393 for the strong, equal and weak conditions, respectively.

**Figure 5.**
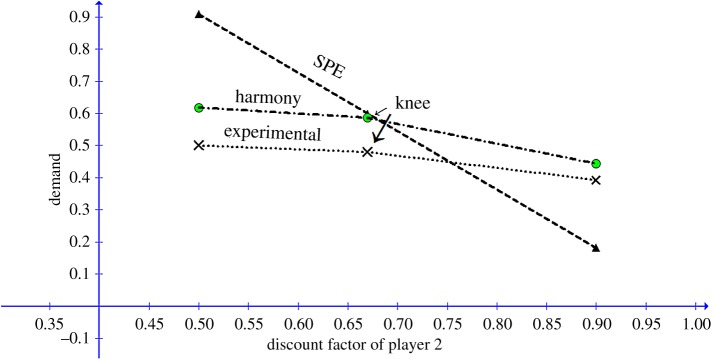
Experimental and theoretical opening demands by P1 for the Weg *et al.* [7] study, as functions of the discount factor of P2.

Figure 5 depicts the experimental results together with the EH and SPE predictions. The mean prediction error for the harmony solution is $\left(1/n)\Sigma_{n}(|d_{o}-d_{p}|\text{/}d_{o}\right)$ × 100 = 19.55%, compared with 53.51% for the SPE solution. As suggested by figure 4, the differences between the harmony predictions and the experimental results for conditions *δ*_2_ = 0.5 and *δ*_2_ = 0.67 are almost equal (0.118 and 0.106, respectively). Thus, we assume that a moderate risk aversion or security level to the utility function of subjects in the role of P1 could lower the harmony prediction error considerably, without affecting the error of the SPE in a similar manner (figure 5).

Strikingly, the discontinuity effect predicted by the model is supported qualitatively by the data, although the observed decline after the knee is less steep in the experimental figure than in the EH prediction (figure 5).

### 4.6. Disadvantageous counter-offers

A qualitative prediction of EH theory states that, in games with *δ*_2_ ≤ *φ* ≈ 0.618, which do not end in agreement in the first period, the demand of P2 in the second will be less than what she had been offered by P1 and was rejected by herself in the first period. Table 5 depicts the percentages of disadvantageous counterproposals in four out of the five investigated experiments.

**Table 5.** Disadvantageous counterproposals in the five discussed studies.

study	observation number	percentage of first offer rejection	percentage of disadvantageous counterproposals
Binmore *et al.* [40]	81	15%	75%
Exp. 1 (*δ* = 0.25)			
Neelin *et al.* [1]			
Exp. 1 (*δ* = 0.25)	120	13%	56%
Exp. 2 (*δ* = 0.50)	45	16%	86%
Exp. 3 (*δ* = 0.34)	165	14%	65%
Ochs & Roth [6]	760	16%	81%
*δ*_2_ = 0.4, 0.6			
Weg *et al.* [7]	108	25%	59.3%
Exp. 1 strong condition			
*δ*_2_ = 0.5			

The study by Binmore *et al.* [2] is not included in the table, because the authors do not provide separate results for disadvantageous counter-offers for games with *δ* ≤ 0.6 and for *δ* > 0.6.

As presented in the table, the percentages of disadvantageous counterproposals by P2 in all the listed experiments were considerably high. Calculation of the weighted average of the percentages of disadvantageous offers across all the listed experiments yields a high percentage of 74.3%, which nicely agrees with the above-stated prediction.

## 5. Concluding remarks

Numerous experimental studies on the ultimatum game and on sequential bargaining games with alternating offers have repeatedly disproved the SPE predictions (for reviews, see [11,55]). The equality principle, sometimes evoked to account for experimental findings, cannot qualify as a theoretical model of bargaining, because it disobeys the rationality principle and it is totally blind to the parameters of the bargaining environment. The performances of theories which compromise the narrow rationality principle by incorporating utilities of interdependent preferences, including ERC [22] and IA theory [23], are far from being satisfactory [2].

While researchers agree that players do not always strive to maximize their monetary pay-offs, we are not aware of a good proposal for an alternative utility function. Notably, Nash's solution of the bargaining problem is based on determining ‘the amount of satisfaction each individual should expect to get from the situation’ [36, p. 155]. Nash stated that rational expectations ‘should be realizable by an appropriate agreement between the two’ and that ‘there should be an available anticipation which gives each the amount of satisfaction he should expect to get’ [36, p. 158]. However, although Nash underscores the view that the concept of anticipation is important in his solution, his derivation is not based on a direct translation of the equality under the satisfaction levels principle.

Under EH theory, we proposed a simple utility function defined as the ratios between a player's actual pay-offs and her aspired pay-offs. We argue that our definition is an intuitive and reasonable formalization of the notion of level of satisfaction. We showed that despite the fact that existing experiments were not designed to test the proposed theory, application of the theory to ultimatum and sequential common pool resource dilemma experiments yielded impressively accurate predictions (see [41]). Here, we demonstrated that theory yields good predictions for several bargaining experiments with alternating offers, using a wide range of equal and unequal discount factors and game horizons. For most investigated experiments, we showed that the theory makes impressive point predictions of the opening demands. The absolute prediction errors for the first experiment in the Binmore *et al.* [40] study, the two experiments by Nileen *et al.* [1] and Game III in the Binmore *et al.* [2] study were 3%, 2% and 7.3%, respectively. Quite strikingly, the predicted discontinuity effect at *δ*_2_ = 0.618 was confirmed by the results of Binmore *et al.* [2] and Weg *et al.* [7], in which high discounting conditions, with *δ*_2_ > 0.618, were included in the experimental design. We note that despite the success of the theory prediction of existing data, future experiments designed particularly for testing the theory are needed. In such research, the salience of the discontinuity point could be enhanced by choosing a more refined sampling of the values of *δ*_2_, particularly in the vicinity of the predicted ‘kink’ point.

It has been noted that the opening demands in bargaining games with alternating offers do not change with changes in the game parameters as sharply as the SPE predicts [2,56]. Our solution captures this behavioural tendency quite well. In fact, EH predicts that if *δ*_2_ ≤ *φ*, where *φ* is the Golden Ratio, the proportional opening demand is predicted to equal *φ* ≈ 0.618, regardless of the value of *δ*_1_and the game's horizon.

Interestingly, EH's derived solutions for the ultimatum game, the CPR dilemma and the sequential bargaining game with alternating offers are all governed by the aesthetically pleasing Golden Ratio. A similar result for the ultimatum game was recently derived independently by Schuster [57], using the method of infinite continued fractions and the Fibonacci numbers. Moreover, independent of our theory and of Schuster's solution of the ultimatum game, Jasso [58,59] wrote that the Golden Ratio has come to light in the context of the justice evaluation function, which compares the actual reward and the just reward. Also, Vermunt [60] stated, without proof, that a fair division (the ‘justice zone’) should lie between the equal split and the inverse GR, which is approximately 0.618. This was, in turn, discussed by Jasso, who wrote that ‘it is possible that further theoretical work could yield a prediction for the golden ratio justice zone’ [61, p. 243].

The Golden Ratio emerges also in other strategic games. For example, in a cyclic four-player bargaining game, a mixed Nash equilibrium exists in which the probabilities of adopting certain strategies are related to the Golden Ratio [62]. In the results, in the cognitive hierarchy model [63], and in some two-player extensive form games in which the pay-offs are drawn at random from a given feasible set, the Golden Ratio emerges for certain parameter settings [64]. The emergence of the Golden Ratio in economic models adds to its numerous appearances in science (e.g. [65–68]) and in aesthetics and the arts [45,69]. The fact that the Golden Ratio plays a key role in human sense of beauty suggests that our tastes for fairness and beauty might be correlated [70,71].

EH theory assumes that individuals in bargaining situations with shrinking pies recognize the fact that they are better off reaching an agreement as early in the interaction as possible and that this requires them to give their counterparts fair enough offers, which they could accept. It is possible, however, that a portion of the bargainers would try to maximize their own pay-offs by squeezing their counterparts into an unfair equilibrium. For example, in the game with equal discount factors and *T* = 2 periods, a self-interested individual in the position of P2, who in period 1 receives a harmonious offer of 1 − *φ *≈ 0.382 of the pie, might behave strategically by rejecting the opening offer if the pie after shrinkage is still larger than what he was offered in period 1; i.e. if *δ* − *ε* > 1 − *φ*, or *δ* > 1 − *φ* + *ε* ≈ 0.382. Future research which discriminates between the players' social preferences might shed more light on the degree of fairness versus gamesmanship in bargaining games. A recent study [72] in which subjects were classified according to the Social Value Orientation (SVO) scale (cf. [73,74]) found that the levels of offers in an alternating offers game were correlated with their SVOs.

In the investigated experiments, sizable minorities of players in the role of second player rejected the opening offers made to them by the first players, and most of them made disadvantageous counter-offers. Ochs & Roth [6], who draw attention to this seemingly unreasonable incident, cited it as evidence that players must be concerned with more than their own monetary pay-offs. Our analysis shows that EH theory, which defines utility in a manner agreeable with Ochs & Roth's conjecture, captures this counterintuitive behaviour quite nicely.

An interesting insight that emerges from the above analysis contradicts the claim by Binmore *et al.* [40], who contended that ‘the one-stage ultimatum game is a rather special case from which it is dangerous to draw general conclusions’ [40, p. 1180]. Our theoretical analysis suggests that the ultimatum game is rather the general case. EH theory predictions, supported by the investigated data, indicate that, as long as the discount factor of P2 is low enough (*δ*_2_ ≤ 0.618), the opening demand of P1 is best predicted by the Golden Ratio, which is also the confirmed prediction for the ultimatum game. In fact, we contend that the game with horizon *T* = 2, used in Binmore *et al.* [40], is a special case, because its simple structure might motivate subjects who know something about game theory to work out the relatively easy backward induction.

Do bargainers behave like gamesmen, who strive to maximize their monetary pay-offs, or like fair men, who opt for the 50–50 split? Taken together, the accumulating results of bargaining experiments indicate that bargainers adhere neither to the SPE equilibrium nor to the equality (50–50) principle. Our theory proposes that a more appropriate answer to the above question would be that rational bargainers strive to maximize their profit while treating their counterparts fairly. By fairness we do not mean equal pay-offs, but equal satisfaction levels from the received pay-offs. We contend that the observed fair demands in bargaining experiments are not motivated by mere benevolence, but by the fear that an unfair proposal might not be satisfying enough to the counterpart and thus might be rejected by her, resulting in more bargaining periods and more severe discounts. It is probable that, in two-period games, in which backward induction is easy to calculate, some bargainers might behave as gamesmen, who tend to maximize their pay-offs; others behave as fair men, who tend to keep their counterpart satisfied; and yet others adhere to some combination of the two tendencies. However, in games with longer horizons in which backward induction is almost impossible, particularly under the pressure of severe discounting, bargainers are more likely to play fair, not due to good manners, but out of necessity.

In deriving the analytical solutions for the discussed games, we relied on rational reasoning to conjecture about the aspiration levels of the interacting players. An interesting extension of our model, which we hope to address in future research, concerns the dynamic aspects of aspirations. By using repeated experimental games or computer simulations, we can test the dynamics of players' aspirations in strategic games, including the two games discussed in this paper. In particular, we would like to investigate the coevolution process of aspirations and decisions in repeated and evolutionary games and test whether the predicted points of harmony for each game will emerge as attractors for the behavioural dynamics.

Another future extension concerns the simplifying assumption of homogeneity. In reality, it is likely that different individuals might adhere to different rules and anchor their aspiration levels on different reference points. Accounting for such epistemic heterogeneity could be executed with an empirical assessment of each individual's aspiration level, given the game structure and the player's position in the game. When measurement of the individuals' aspiration levels is unreliable, costly or difficult to perform, the predictability power of the proposed theory could be enhanced by assuming that the players’ aspiration levels are sampled from a theoretical probability distribution. An example of using an underlying probability distribution of aspiration levels to infer about offers was discussed in Suleiman [41] for the case of the ultimatum game, in which we assumed that the proposers' aspiration levels fall within the range between 1 (perfect rationality) and 1 − *α* (bounded rationality), where *α* is a uniformly distributed security factor. In Suleiman [41], we derived a general term for the harmony solution as a function of the security level *α*. For the special case in which the proposer aspires to get any amount between 100% and 75% of the entire amount, the derived solution yields a mean harmonious offer of approximately 0.40, which is less than 5% higher than 0.382, the predicted offer for *α* = 0. Another possible source of heterogeneity might stem from individual differences in risk preferences. For the case of power utility functions, under plausible assumptions, we derived from Suleiman [41], a general expression for the harmony offer as a function of the ratio between the risk indices of the proposer and the responder. With a ratio ranging between 1 (equal risk indices) and 2 (proposer is twice riskier), we found that the mean predicted offer is approximately 0.402, which is approximately 5% less than the 0.382 predicted under the linearity assumption. Thus, it seems fair to conclude that the Golden Ratio solution, which emerged as another solution within the bargaining game, is a relatively robust prediction under reasonable variations in the proposers’ security levels and the players' risk preferences.

Throughout our analysis, we assumed that the players’ utilities are functions only of the ratio between their actual monetary pay-offs and their aspired pay-offs. Obviously, this is a simplification that ignores non-monetary utilities, such as pride, self-worth and revenge. Such motivations, not discussed in this paper, are manifest in ultimatum bargaining and in other strategic games. For example, a study on the impunity game, in which a rejection by the responder does not reduce the proposer's demand, showed that a substantial proportion of low offers are rejected [75]. It was argued that such behaviour is mediated by emotions that restrain people from responding to the immediate incentives and protect their reputation, which may be more valuable in the long run [76]. In a variant of the impunity game, termed the ‘take-it-or-leave-it’ game [77], rejection of an offer by the responder granted the proposer the entire amount. Thus, not only can the responder not punish the proposer, but also by rejecting her low offer, she can actually benefit herself even more. We found that a sizable minority of responders still reject low offers. Accepting low offers was found to be associated with the desire for profit maximization, and rejecting similarly low offers was associated with the desire to maintain self-worth. We concluded that rejection of low offers in the investigated game, as in many significant real-life situations, could be explained as costly signals aimed at protecting the attributes of symbolic capital, such as self-worth, status and prestige. Chivalry and benevolence also play a role in the proposers' motivations, causing some to offer even more than they keep for themselves.

Our analysis of bargaining games, including the ultimatum game, suggests that first players might allocate fairly, with neither altruistic motives nor consideration of social mechanisms that promote fair behaviour. In this aspect, our model has a clear advantage over models like ERC, IA, envy and empathy-based statistical physics models of human behaviour [37–39], because all the aforementioned theories and models presuppose empathetic or envious motives towards other players, which entail the assumption of fairness. By contrast, EH theory prescribes fairness as a predicted outcome reached by rational players, who strive to maximize their utilities, as defined here. Thus, the proposed model carries the message that fair treatment is beneficial not only for the recipient, but also for the provider. This message could prove important in applying the theory to real-life situations, like in bargaining and negotiations between parties, when the negotiated goods depreciate with time. Such situations include negotiations between companies, between insurance companies and their clients, between an employer and a workers' union, and between parents about housework and childcare. In another paper, we investigated the important issue of salaries. Comparison between actual mean salaries of senior and junior employee in high-tech and non-high-tech professions, from developed countries with high gross national income (GNI) and developing countries with low GNI, revealed that, for developed countries, the average ratios of the juniors' salaries to the total salaries are almost the same for the high- and low-tech professions (≈0.37) and are only slightly below the Golden Ratio prediction of ≈0.38. On the other hand, for the low-tech professions, the mean ratio of the juniors’ salaries is approximately 28%, significantly lower than the mean ratio of the seniors' salaries.

Quite interestingly, the solutions derived from statistical physics, prescribing collective behaviour near the phase transition points of particles’ interaction [37–39], fit well with the Golden Ratio solution derived in this paper, which was in fact inspired by my recently proposed Information Relativity theory (e.g. [78–81]). This theory predicts that matter enters into a quantum phase transition at velocities equalling *φ c*, where *c* is the velocity of light and *φ* is the Golden Ratio (approx. 0.618). Experimentally, Coldea *et al.* [68] reported a similar result. By applying a magnetic field at right angles to an aligned chain of cobalt niobate atoms, they demonstrated that the cobalt entered a quantum critical state, and that just below the critical field, the spin dynamics revealed a fine structure, with two sharp modes at low energies, in a ratio that is close to the Golden Ratio.

## Data accessibility

This article has no additional data.
